# Protein Aggregation of NPAS3, Implicated in Mental Illness, Is Not Limited to the V304I Mutation

**DOI:** 10.3390/jpm11111070

**Published:** 2021-10-23

**Authors:** Bobana Samardžija, Aristea Pavešić Radonja, Beti Zaharija, Mihaela Bergman, Éva Renner, Miklós Palkovits, Gordana Rubeša, Nicholas J. Bradshaw

**Affiliations:** 1 Department of Biotechnology, University of Rijeka, 51000 Rijeka, Croatia; bobana.samardzija@biotech.uniri.hr (B.S.); beti.zaharija@biotech.uniri.hr (B.Z.); mihaela.bergman@student.uniri.hr (M.B.); 2 Psychiatry Clinic, Clinical Hospital Centre Rijeka, 51000 Rijeka, Croatia; aristeapr@medri.uniri.hr (A.P.R.); gordana.rubesa@medri.uniri.hr (G.R.); 3 Faculty of Medicine, University of Rijeka, 51000 Rijeka, Croatia; 4 Human Brain Tissue Bank & Laboratory, Semmelweis University, 1094 Budapest, Hungary; renner.eva@med.semmelweis-univ.hu (É.R.); palkovits.miklos@med.semmelweis-univ.hu (M.P.)

**Keywords:** blood serum, major depressive disorder, insular cortex, mental illness, neuronal PAS protein 3 (NPAS3), post-mortem brain tissue, protein aggregation, proteinopathy, schizophrenia

## Abstract

An emerging phenomenon in our understanding of the pathophysiology of mental illness is the idea that specific proteins may form insoluble aggregates in the brains of patients, in partial analogy to similar proteinopathies in neurodegenerative diseases. Several proteins have now been detected as forming such aggregates in the brains of patients, including DISC1, dysbindin-1 and TRIOBP-1. Recently, neuronal PAS domain protein 3 (NPAS3), a known genetic risk factor for schizophrenia, was implicated through a V304I point mutation in a family with major mental illness. Investigation of the mutation revealed that it may lead to aggregation of NPAS3. Here we investigated NPAS3 aggregation in insular cortex samples from 40 individuals, by purifying the insoluble fraction of these samples and testing them by Western blotting. Strikingly, full-length NPAS3 was found in the insoluble fraction of 70% of these samples, implying that aggregation is far more widely spread than can be accounted for by this rare mutation. We investigated the possible mechanism of aggregation further in neuroblastoma cells, finding that oxidative stress plays a larger role than the V304I mutation. Finally, we tested to see if NPAS3 aggregation could also be seen in blood serum, as a more accessible tissue than the human brain for future diagnosis. While no indication of NPAS3 aggregation was seen in the serum, soluble NPAS3 was detected, and was more prevalent in patients with schizophrenia than in those with major depressive disorder or controls. Aggregation of NPAS3 therefore appears to be a widespread and multifactorial phenomenon. Further research is now needed to determine whether it is specifically enhanced in schizophrenia or other mental illnesses.

## 1. Introduction

Schizophrenia and major depressive disorder are severe, and in many instances chronic, mental illnesses, for which diagnosis can only be achieved through structured clinical interview, supplemented by medical scales for determining symptom presence and severity. Genetics studies have been successful in identifying a large number of haplotypes associated with both schizophrenia [1,2,3] and major depressive disorder [4] at a genome-wide level. All variants detected in this manner, however, have been of modest individual effect sizes, requiring a polygenic approach for future clinical usage. As a complementary approach, we have proposed also investigating protein homeostasis in patients with major mental illness, and in particular, looking at proteins that may form insoluble aggregates specifically in the brains of patients with chronic conditions [5,6]. Such an approach is partially analogous to the presence of similar aggregates in many neurodegenerative disorders. Aggregates in mental illness would not be expected to be neurotoxic, given the lack of known cell death in these conditions [5].

To date, several proteins have been published which may form such aggregates in patients with chronic mental illness, one of that is neuronal PAS protein 3 (NPAS3), a brain-expressed transcription factor. NPAS3 was originally implicated in mental illness due to a chromosomal translocation that disrupted the *NPAS3* gene in a mother with schizophrenia, as well as in her daughter, who had schizophrenia and mild learning difficulties [7,8]. Variants in *NPAS3* have since been associated with several major mental illnesses [9,10,11] and response to an antipsychotic [12], while mice lacking the *Npas3* gene also display behavioral abnormalities [13]. More recently, a small family was reported in which a rare V304I mutation was present in three individuals with schizophrenia and one with major depressive disorder [14]. When analyzed, using both purified recombinant protein and exogenous protein in cell culture, the V304I mutant form of NPAS3 was found to be insoluble. This contrasted with the wild-type protein, which was soluble in the same assays, indicating that this mutation is capable of causing aggregation of NPAS3 [15], at least in these experimental systems. To the best of our knowledge, however, this has not yet been confirmed in human brain samples, nor has it been established whether NPAS3 aggregation is a phenomenon beyond this specific family.

This mutation is extremely rare (minor allele frequency 0.0001–0.0004 [16]), and is, to the best of our knowledge, the only known instance of a specific mutation driving protein aggregation in mental illness. Other proteins have been observed to aggregate in the brains of patients with mental illness [6,17,18,19], with each being found in a specific subset of individuals (typically 10–20% of patients), but the underlying pathological events behind this are unclear [5].

While the study of protein aggregation in mental illness is still relatively new, it is a well-established phenomenon of many neurodegenerative diseases. The presence of proteins aggregating in a distinct subset of mental illness patients appears to be analogous to conditions such as ALS or frontotemporal lobe dimension (FTD), in which patients typically display aggregation of one of several proteins, such as TDP-43 or FUS. Aggregation of these proteins is in turn driven by a variety of familial and spontaneous mutations, as well as environmental factors [20,21,22].

We therefore set out to determine whether aggregation of NPAS3 may be a general phenomenon of major mental illness, and if it is present in a greater subset of individuals than would be predicted by the rare V304I mutation.

## 2. Materials and Methods

### 2.1. Human Brain Samples

Samples of short post-mortem delay (1–10 h) human brains, donated through the Hungary-wide Lenhossék Program, after obtaining consent from the family or legal permission, were dissected using a punch technique [23,24]. The samples were kept frozen during the micropunch procedure and stored at −70 °C. For this study, samples from the insular cortex of 40 individuals were used: 14 from victims of suicide, 14 from control individuals, 6 from patients with depression and 6 from patients with Alzheimer’s disease. Demographic information can be found in Supplementary Table S1. One additional sample (from a suicide victim) was excluded from the study as the physical size of the sample was too small for the analysis required. All data was anonymized and further encoded, such that the researcher performing all experimental procedures was unaware of the diagnostic status of each brain sample. Data was decoded only after quantification of data.

### 2.2. Human Blood Samples

Blood serum samples were collected from 150 individuals at the Clinical Hospital Centre Rijeka. Fifty each from patients with schizophrenia or major depressive disorder were recruited through the Psychiatry Clinic, while 50 from healthy control individuals were recruited through the Clinical Institute of Transfusion Medicine. Control individuals were excluded if their medical records showed any incidence of psychiatric or neurological disorders. Patients were diagnosed according to either ICD-10 [25] or DSM-V criteria [26]. All participants were 18–72 years old and Croatian. Further demographic details can be found in Supplementary Table S2. In total, patients required 60–100 min of contact time, less for control individuals. All data was anonymized and further encoded, such that the researcher performing all experimental procedures was unaware of the diagnostic status of each serum sample. Data was decoded only after quantification of data.

### 2.3. Insoluble Protein Purification from Brain

The procedure for purifying the insoluble protein fraction of brain samples was adapted from one published previously [6]. Brain samples were homogenized to a concentration of 10% w/v in Purification Buffer (50 mM HEPES pH 7.5, 250 mM sucrose, 5 mM magnesium chloride, 100 mM potassium acetate, 2 mM PMSF) containing 1% Triton X-100 and protease inhibitor cocktail. Homogenate was centrifuged at 20,000× *g* for 20 min. The supernatant was discarded and the pellet was resuspended in the same buffer and centrifuged again, using the same conditions. The pellet was then resuspended in Purification Buffer containing 1.6 M sucrose and 1% Triton X-100, and then centrifuged at 130,000× *g* for 45 min. The pellet was then resuspended in Purification Buffer containing 1 M NaCl plus DNaseI and incubated for 16 h at 4 °C. The samples were then centrifuged three more times at 130,000× *g* for 45 min, with the pellet being resuspended the first two times in 10 mM HEPES pH 7.5, 5 mM EDTA, 0.5% sarkosyl. After the final spin, the remaining insoluble pellet was resuspended in 2× SDS-PAGE loading buffer for blotting. All centrifugations were performed on a Sorval MTX ultracentrifuge with a S140-AT fixed angle rotor (Thermo Fisher Scientific, Waltham, MA, USA) and thick-walled 11 × 34 mm polyallomer tubes (Science Services, Munich, Germany). The entire procedure was conducted at 0–4 °C. A researcher who was blinded to the diagnostic status of each sample performed the purification procedure and subsequent Western blot analysis.

### 2.4. Serum Samples and Insoluble Protein Purification from Serum

Blood samples were collected in Vacuette CAT Serum Sep Clot Activator Tubes (Greiner Bio-One, Kremsmünster, Austria) and immediately centrifuged to separate out the serum. Samples were then immediately frozen and stored at −80 °C until further processing. Purification of the insoluble protein fraction was adapted from existing protocols [19,27], and optimized for use with blood serum. Serum samples were thawed and mixed with an equal volume of 50 mM sodium phosphate pH 7.4/500 mM sucrose/200 mM potassium acetate/40 mM magnesium chloride/10% Triton X-100/2 mM PMSF/100 units/mL DNaseI and incubated on ice for one hour. After this time, 3% of the sample was removed and stored as “whole serum”. The remainder was centrifuged at 20,000× *g* for 1 h. The supernatant was then removed and the pellet resuspended in 50 mM sodium phosphate pH 7.4/1.5 M sodium chloride/500 mM sucrose/200 mM potassium acetate, before being centrifuged again at 20,000× *g* for 1 h. This procedure was repeated with two further buffers: 50 mM sodium phosphate pH 7.4/1.6 M sucrose/200 mM potassium acetate and 50 mM sodium phosphate pH 7.4/200 mM potassium acetate/1% NP-40/0.2% sarkosyl. The final pellet (the “insoluble protein fraction”) was resuspended in SDS-PAGE loading buffer. The entire procedure was conducted at 0–4 °C. A researcher who was blinded to the diagnostic status of each sample performed the purification procedure and subsequent Western blot analysis.

### 2.5. Plasmids

Plasmids encoding NPAS3 with HA-tags [28] were gifts from Dr. Fred Berry (University of Alberta, Edmonton, Canada). A plasmid encoding a fragment of TRIOBP-1 [29] was a gift from Prof. Dr. Carsten Korth (Heinrich Heine University, Düsseldorf, Germany). Gateway entry vectors encoding human NPAS3 and dysbindin-1A came from the ORFeome Collaboration [30] (clones HsCD00080332 and HsCD00510719 respectively from the DNASU Plasmid Repository [31], Tempe, AZ, USA). Additional fragments of NPAS3 were subcloned into pENTR1A no ccDB [32] (Eric Campeau, AddGene clone 17398, Watertown, MA, USA). Genes in Gateway entry vectors were transferred into pdcDNA-FlagMyc (B. Janssens, clone LMBP 4705 from the BCCM/LMBP Plasmid Collection, Zwijnaarde, Belgium), pDEST-CMV-N-mCherry [33] (Robin Kettler, Addgene clone 123215) or pETG10A (A. Geerlof, EMBL, Heidelberg, Germany) using LR Clonase II (Thermo Fisher Scientific, Waltham, MA, USA, 656120). All plasmids were confirmed by sequencing. Details of plasmids and primers are in Supplementary Tables S3 and S4.

### 2.6. Mammalian Cell Culture

SH-SY5Y human neuroblastoma cells (Deutsche Sammlung von Mikroorganismen und Zellkulturen, Braunschweig, Germany) and HEK293 kidney cells (American Type Culture Collection, Manassas, VA, USA) were cultured in DMEM/F-12 and DMEM media, respectively, supplemented with 10% fetal calf serum, pennicilin, streptomycin and non-essential amino acid solution (all media from Thermo Fisher Scientific, Waltham, MA, USA). Cells were transfected with Metafectene (HEK293) or Metafectene Pro (SH-SY5Y) according to manufacturer’s instructions (Biontex, Munich, Germany). For some experiments, cells were treated with 0.5 mM sodium arsenide (or vehicle control) to induce stress, immediately before being fixed for downstream analysis.

### 2.7. Insoluble Protein Purification from Mammalian Cell Lysates

Transfected HEK293 cells were lysed for 30 min at room temperature on a rotary wheel in 50 mM HEPES pH 7.5/250 mM sucrose/12.5 mM magnesium chloride/100 mM potassium acetate/2 mM PMSF/1.5% NP-40/0.3% sarkosyl/20 mM calcium chloride, containing proteinase inhibitors and DNaseI, after which some of the lysate was stored. The remainder was then mixed with 0.5 volumes of 50 mM HEPES pH 7.5/2.3 M sucrose/1% NP-40/0.2% sarkosyl and ultracentrifuged at 130,000× *g* for 1 h. The supernatant was then discarded. The pellet was then put through three further cycles: in each cycle, the pellet was resuspended in a different buffer, ultracentrifuged as described above, and finally had its ensuing supernatant discarded. The buffers used for these cycles were, in order: (1) 50 mM HEPES pH 7.5/1.6 M sucrose/1% NP-40/0.2% sarkosyl, (2) 50 mM HEPES pH 7.5/1.5 M NaCl and (3) 50 mM HEPES pH 7.5/1% NP-40/0.2% sarkosyl. The final pellet, representing the insoluble protein fraction, was resuspended in SDS-PAGE loading buffer. Procedure was performed at 0–4 °C and based on a previously published protocol [34].

### 2.8. Recombinant Protein Expression in Bacteria

Plasmids encoding human proteins (or protein fragments) were transformed into BL21(DE3) pLysS competent cells (Promega, Madison, WI, USA) and grown in 2xYT media with ampicillin and chloramphenicol at 37 °C. Cultures growing at an optical density of OD_600_ = 0.6 had protein expression induced with 1 mM IPTG (UBPBio, Aurora, CO, USA) for 3 h at 30 °C.

### 2.9. Western Blotting and Analysis

Protein samples (from brain, blood serum or cultured cells) were run on bis-acrylamide gels, and then transferred to PVDF membranes using a Trans-blot Turbo Transfer System (Bio-Rad, Hercules, CA, USA). Sample loading was verified using 0.5% Ponceau S/2% acetic acid. Membranes were blocked in Aqua Block (Abcam, Cambridge, UK, for blood samples: two hours at room temperature, for brain samples: overnight at 4 °C) or PBS/0.05% Tween 20/5% milk powder (for cultured cells, one hour at room temperature). They were then stained with primary antibodies against β-actin (TA811000, Origene, Rockville, MD, USA), dysbindin-1 (HPA028053, Atlas Antibodies, Broma, Sweden), Flag (F1804, Merck, Darmstadt, Germany), HA (H3663, Merck), NPAS3 (PK-AB718-4107, PromoCell, Heidelberg, Germany, plus for a verification experiment: 4107, ProSci, Poway, CA, USA) and/or TRIOBP-1 (HPA019769, Atlas Antibodies) in PBS/0.05% Tween 20. After washing in the same buffer, a secondary antibody was applied (31430 or 65-6120, Thermo Fisher Scientific, Waltham, MA, USA). Protein was visualized using ECL Prime Western Blotting Detection Reagent (GE Healthcare, Chicago, IL, USA, for clinical samples) or Pierce ECL Western Blotting Substrate (Thermo Fisher Scientific, Waltham, MA, USA, for cultured cells) on a ChemiDoc MP Imaging System (Bio-Rad). Membranes were visualized and quantified (where necessary) using Image Lab software (Bio-Rad).

Antibodies used to detect TRIOBP and dysbindin-1 in this serum have been characterized and used previously [19,29,35]. These, plus the NPAS3 antibody used for analysis of blood, were additionally tested for specificity (Supplementary Figure S1).

### 2.10. Immunocytochemistry and Microscopy

Cells were fixed with PBS/4% paraformaldehyde for 15 min and then permeabilized with PBS/1% Triton X-100 for 10 min. Cells were washed three times with PBS, then blocked in PBS/10% goat serum for 45 min. Cells were then stained with anti-Flag or anti-HA antibody in PBS for 4 h, before being washed 4 more times (over 15 min). Cells were then stained with secondary antibody (A11037, Thermo Fisher Scientific, Waltham, MA, USA), fluorescently labelled phalloidin (PHDG1-A, Cytoskeleton, Denver, CO, USA) and DAPI (Merck) for 1 h, before 4 final washes (as above). Slides were mounted onto glass coverslips using Fluoroshield (Merck) and viewed on an IX83 fluorescent microscope (Olympus, Shinjuku, Japan), using an Orca R2 CCD camera (Hamamatsu Photonics, Hamamatsu, Japan) and CellSens software (Olympus, Shinjuku, Japan). When performing quantified assays, transfections, immunocytochemistry and microscopy were performed by a researcher who was blinded as to which plasmid was being introduced on each cover slip. This data was decoded only after cells had been viewed and counted.

### 2.11. Statistical Analysis

Statistical analysis was performed in JASP [36]. Comparisons of two or more groups with one variable were performed by one-way ANOVA with Brown-Forsythe homogeneity correction (considered significant if F > F_crit_ and *p* < 0.05), followed by Tukey’s post hoc test if three or more groups were involved (*p*_tukey_ < 0.05 was considered as significant). For analysis of blinded cell culture experiments a nominal value of 0.1% was inserted into one sample if all replicates of a sample equaled zero. Comparisons of two groups with two variables were done by multivariate or two-way ANOVA (considered significant if F > F_crit_ and *p* < 0.05) as stated in the text.

## 3. Results

### 3.1. Insoluble NPAS3 Is Common in Human Post Mortem Insular Cortex

Previous studies looking for evidence of protein aggregation in major mental illness have typically been performed by purifying an insoluble protein fraction from brain samples of patients and control individuals, with specific insoluble proteins being found in 10–30% of cases [6,17,18]. Cell and animal models have then been used to strongly imply that these insoluble species do indeed represent aggregates [17,18,19,27,37,38,39]. We therefore performed similar studies in insular cortex samples from suicide victims (*n* = 14), patients with depression (*n* = 6) and control individuals (*n* = 18). Patients with Alzheimer’s disease (*n* = 6) were also included for context. The suicide victims and control individuals had similar sex balances, although the suicide victims were on average 15 years younger at time of death (details in Supplementary Table S1). Intervals between death and sample collections did not significantly differ between the diagnostic categories.

**Figure 1:**
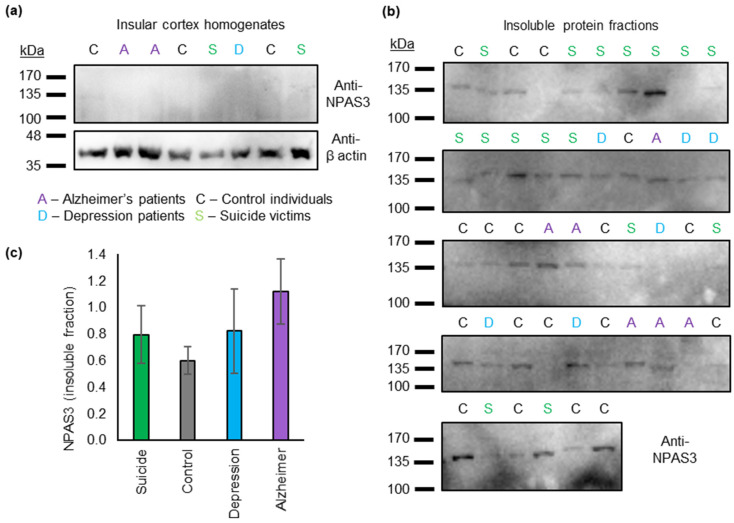
Insoluble (aggregating) NPAS3 in 46 insular cortex samples. (**a**) Sample Western blot of whole cortex homogenates, showing no discernable NPAS3, while actin is visible. (**b**) Blots of the insoluble protein fractions, which are strongly enriched for aggregating proteins, derived from all insular cortex samples. (**c**) Quantification of the amount of NPAS3 in each sample, grouped by diagnostic status. There were no statistically significant effects, although there is a trend difference between Alzheimer’s disease patients and control individuals. NPAS3 signal in each category is shown relative to the average NPAS3 signal over all samples.

Levels of NPAS3 in the whole brain sample were extremely low (Figure 1a), which is to be expected given that NPAS3 is principally expressed in early development, and its mRNA levels diminish in the brain after birth [40]. In purified fractions of the brain samples that had been strongly enriched for insoluble (including aggregating) protein, the major NPAS3 species seen was at 130 kDa, corresponding to the full-length protein (Figure 1b). Strikingly, full-length NPAS3 was seen in the insoluble fractions of approximately three-quarters of the brain samples examined, suggesting that NPAS3 aggregation is far more common than would be expected based on the rare V304I mutation. The presence of insoluble NPAS3 was present across all diagnoses and showed no specificity to any diagnoses (Figure 1c, ANOVA, *p* = 0.24, F < F_crit_, df = 3, relative levels of NPAS3 by diagnostic status were suicide: 0.78 ± 0.22, control: 0.60 ± 0.11, depression: 0.82 ± 0.25, Alzheimer’s: 1.20 ± 0.32).

NPAS3 aggregation, previously implicated in schizophrenia, therefore appears to be much more widespread than expected. There was no significant association between NPAS3 aggregation and suicide or any other condition tested. Curiously, in spite of the low number of samples used, there was a trend relationship between NPAS3 aggregation and a diagnosis of Alzheimer’s disease, which should be studied with a larger sample.

### 3.2. NPAS3 Aggregation in Cultured Cells Can Derive from Oxidative Stress and the V304I Mutation

**Figure 2:**
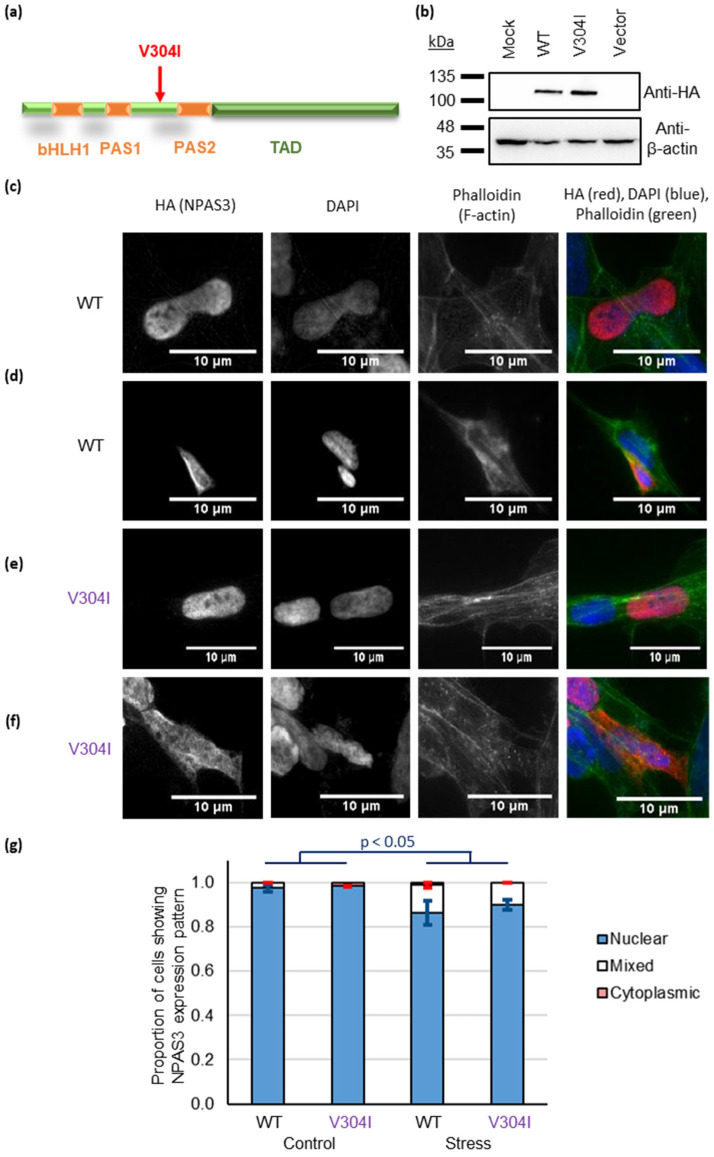
Mislocalization of NPAS3 to the cytoplasm does not require the V304I mutation, as determined in SH-SY5Y neuroblastoma cells. (**a**) One-dimensional schematic of the NPAS3 protein, showing major domains and the location of the V304I mutation. Abbreviations: bHLH: Basic helix-loop-helix domain, PAS: Per-Amt-Sim domain, TAD: C-terminal activator domain. (**b**) Expression of NPAS3 wild type (WT) and V304I mutant constructs, confirmed by Western blotting in HEK293 cells. Negative controls are mock transfection and transfection with empty vector only, (**c**–**f**) Typical localization patterns of NPAS3, both wild type and mutant V304I when over-expressed in SH-SY5Y cells, with each showing a mixture of nuclear and cytoplasmic localization: (**c**) WT in the nucleus, (**d**) WT in the nucleus and cytoplasm, (**e**) V304I in the nucleus, (**f**) V304I in the cytoplasm. The nucleus (DAPI) and actin cytoskeleton (phalloidin) are shown for context. (**g**) Quantification of the localization patterns from parts c-f in a blinded experiment. Cells were transfected with either WT or V304I NPAS3 and then exposed to either sodium arsenide (which induces oxidative stress) or an equivalent volume of DMSO as a control. 8 experimental replicates were performed for each combination of plasmid/stress status, with 7–10 cells being counted in each replicate.

The presence of insoluble NPAS3 (which likely represents NPAS3 aggregation) in such a large number of patients was surprising given that aggregation of NPAS3 has only previously been associated with the V304I mutation (Figure 2a), and the rarity of this mutation means that it is almost certainly not present in any of our 40 brain samples [14,15,16]. We therefore decided to investigate NPAS3 in a cell culture model, to determine whether this mutation is required for aggregation.

Previous experiments by Nucifora et al. have demonstrated that this V304I mutation makes NPAS3 more insoluble [15], which suggests it to be misfolding and forming insoluble aggregates. We adopted a different, but complementary, approach of looking for aggregation visually using immunocytochemistry in cultured cells. Specifically, in the case of other nuclear proteins implicated in aggregation, such as TDP-43 (TAR DNA-binding protein 43) in ALS [41], the early stages of aggregate formation interfere with the transport of the protein into the nucleus, causing it to instead be found in the cytoplasm of the cell. Once there, it may also go on to form large visible aggregate structures, however expression in the cytoplasm alone is sufficient to cause negative effects on the cell, without the need for visible inclusion bodies or other aggregate structures [42]. We therefore used cytoplasmic expression of NPAS3 as a surrogate for its misfolding and aggregation, while also looking for visible aggregates.

Plasmids expressing full-length NPAS3, either wild-type (WT) or V304 (Figure 2b), were therefore expressed in SH-SY5Y human neuroblastoma cells. Both versions of NPAS3 were found principally in the nucleus (Figure 2c,e and Supplementary Figure S2a,b,e,f), as would be expected, however in some cells they were also seen to be in the cytoplasm (Figure 2d,f, Supplementary Figure S2c,d,g,h). Similar results were seen at 24, 48 or 72 h after transfection (Supplementary Figure S3), or when wild-type NPAS3 was expressed using two alternative plasmid vectors (Supplementary Figure S4). NPAS3 wild type and V304I plasmids were also expressed in HEK293 cells, that were then subjected to an insoluble protein purification protocol, analogous to that used for the brain samples. A small proportion of both wild type and V304I mutant NPAS3 was confirmed to be consistently insoluble over three experiments, however, there was no difference in levels of insolubility between them (Supplementary Figure S5).

To confirm the principle that factors other than the V304I mutation could affect cytoplasmic localization, we looked at the effect of oxidative stress on early aggregation in SH-SY5Y cells. Oxidative stress was applied using sodium arsenide, which is established to induce aggregation of TDP-43 [43], and the effects of this stress on protein localization were investigated. Specifically, cells were transfected for 24 h, then treated with either sodium arsenide or an equivalent volume of DMSO, for 15 min, before being fixed. After only this short time, there was a significant effect of stress status on nuclear localization (Figure 2g, two-way ANOVA, *p* < 0.05, F > F_crit_, df = 1), with cells that had been treated with sodium arsenide being less likely to show NPAS3 expression exclusively in the nucleus (proportion of cells in the nucleus: WT with stress 86.3 ± 5.4%, V304I with stress 90.0 ± 2.3%, WT without stress 97.5 ± 1.5%, V304I without stress 98.4 ± 0.2%). There was no significant effect of mutation status, nor a significant interaction between mutation and stress status. From this, we can conclude that, at least within this system, there are different mechanisms that can govern the cytoplasmic localization of NPAS3, and it is therefore likely that NPAS3 aggregation can arise from environmental stressors, potentially in addition to genetic ones.

### 3.3. NPAS3 Aggregation in Cultured Cells Is Driven by Its PAS1 Domain

**Figure 3:**
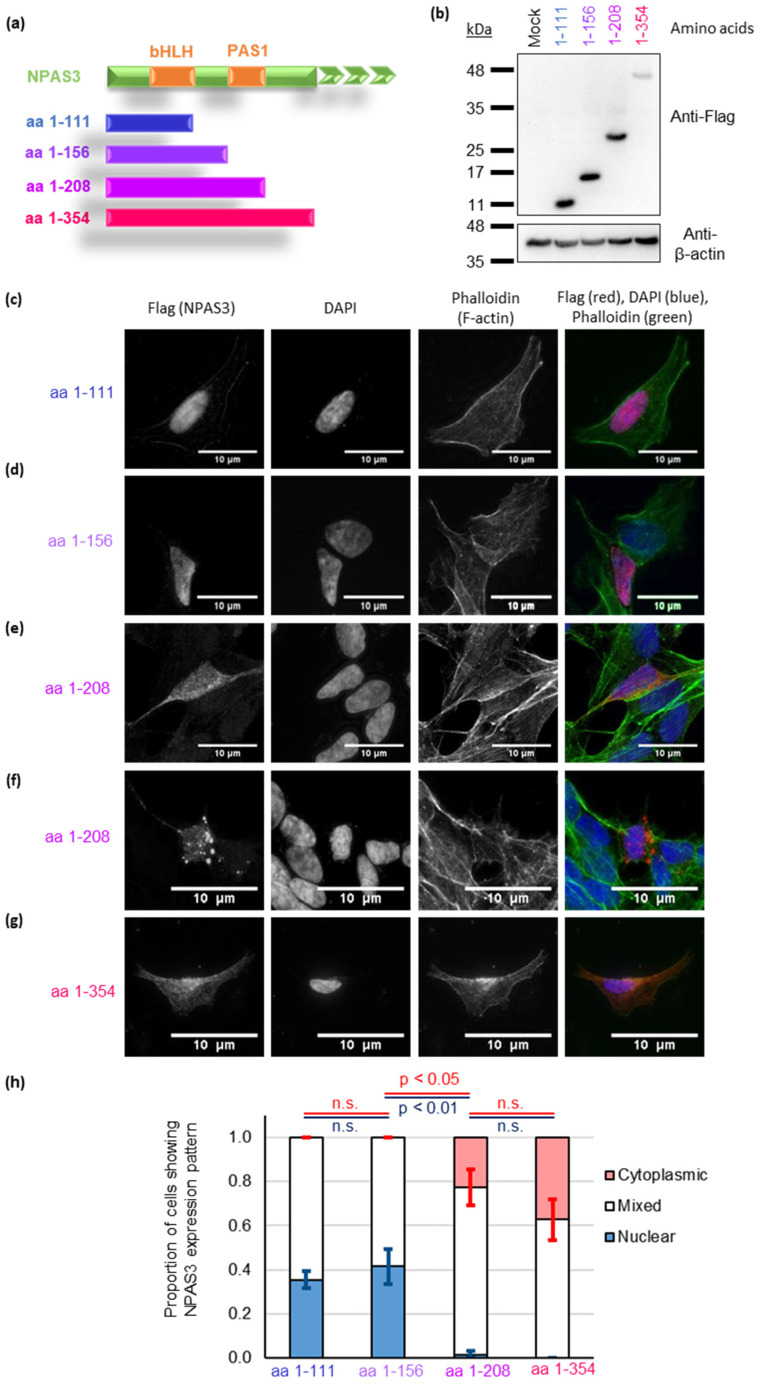
The PAS1 domain is critical for NPAS3 aggregation, as determined by the expression of Flag-tagged NPAS3 fragment in SH-SY5Y cells. (**a**) Schematic of the fragments of NPAS3 expressed. (**b**) Expression of the flag-tagged NPAS3 fragments confirmed by Western blotting in HEK293 cells. A mock transfection with no plasmid is used as a negative control. (**c**–**g**) Typical localization patterns of these NPAS3 fragments when over-expressed in SH-SY5Y cells: fragments containing amino acids 1-111 (**c**) or 1-156 (**d**) (bHLH domain with or without linker region) are seen in the nucleus, while those containing amino acids 1-208 (**e**,**f**) or 1-354 (**g**) (bHLH and PAS1 domains, with or without linker region) are seen in the cytoplasm and in some instances form discernable aggregates. The nucleus (DAPI) and actin cytoskeleton (phalloidin) are shown for context. All amino acid numbers refer to the 933 long amino acid splice variant of human NPAS3 (NP_001158221.1). (**h**) Quantification of the localization patterns of these aggregates in a blinded assay (5–6 experimental replicates per plasmid, with 4–12 cells being counted in each). n.s.: not significant.

We have previously determined that aggregation of TRIOBP-1, another protein implied to aggregate in mental illness, occurs specifically through one structural region [29], and it is therefore possible that aggregation of non-mutant NPAS3 is also driven by a specific structural feature. Four NPAS3 constructs were therefore cloned, all starting at the N-terminus of the protein but truncated from the C-terminus (Figure 3a, see also Figure 2a for the whole domain structure of NPAS3). These variously encoded a basic helix-loop-helix (bHLH) region, the PAS1 domain and unstructured regions, while adding a flag tag to the N-terminus (Figure 3b).

The two shorter plasmids, encoding the bHLH domain plus one or two unstructured regions, expressed fragments of NPAS3 with principally nuclear localization (Figure 3c,d, Supplementary Figure S6a,b,f,g). Intriguingly, the two longer plasmids, which added the PAS1 domain and in one case an additional unstructured region, instead showed prominent protein localization in the cytoplasm (Figure 3e,g, Supplementary Figure S6c–e,h–j). In some cells, these constructs also showed discernible aggregate-like structures (Figure 3f, typical of 14% of cells expressing this construct), which were not seen in any cells expressing the constructs that lacked the PAS1 domain.

To verify this, a further blinded quantification experiment was performed. There were significant effects of plasmid identity on both nuclear and cytoplasmic localization (Figure 3h, one-way ANOVA, p < 0.001 and p = 0.005 respectively, F > F_crit_, df = 3. Cells with nuclear localization only: 1-111: 35.3 ± 3.8%, 1-156: 41.4 ± 8.0%, 1-208: 1.5 ± 1.5%, 1-354: 0%. Cells with cytoplasmic localization only: 1-111: 0%, 1-156: 0%, 1-208: 22.6 ± 8.2%, 1.354: 37.3 ± 9.2%). Localization was seemingly dependent on the PAS1 domain. Post hoc analysis showed that the two plasmids lacking this domain had significantly more nuclear localization than the two containing it (p_tukey_ < 0.001 in all cases) as well as less cytoplasmic localization (p_tukey_ ≤ 0.05 in all cases). There were no significant differences in localization between the two fragments lacking the PAS1 domain, nor between the two containing the PAS domain. It, therefore, appears that aggregation of NPAS3, in the absence of the V304I mutations, is dependent on the PAS1 domain, at least in this experimental system.

### 3.4. NPAS3 Is Also Found in Blood Serum, and Shows Expression Differences in Patients with Schizophrenia

While the presence of NPAS3 aggregates in the brain is interesting from a mechanistic standpoint, it does not represent a practical biomarker for diagnosis. We therefore asked whether similar insoluble or aggregating NPAS3 might also exist in more accessible tissue, such as the blood. We therefore adapted existing protocols for purifying insoluble aggregation, for use with blood serum. These were applied to blood serum samples from 50 patients with depression, 50 with schizophrenia and 50 from control individuals. While NPAS3 was not detected in any of the insoluble fractions, it was visible in the unfractionated serum of several individuals, which is a surprise given that NPAS3 is canonically considered to be principally a neuronal protein, expressed predominantly in the brain [7,44].

**Figure 4:**
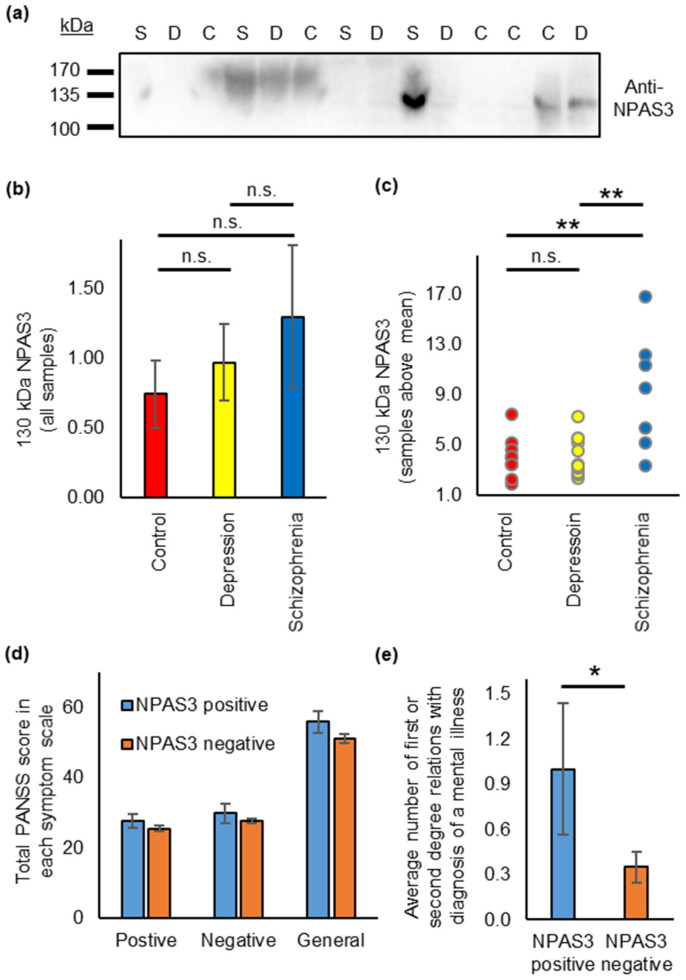
Raised levels of NPAS3 in the serum of a subset of schizophrenia patients. (**a**) Sample Western blot of whole serum samples from patients with schizophrenia (S) or depression (D), as well as control individuals (C). Stained with an anti-NPAS3 antibody, showing 14 samples (all samples in Supplementary Figure S7). Each lane was loaded with exactly 10 μL of serum. (**b**) Amount of 130 kDa NPAS3 detected in all serum samples, normalized to the mean over the entire experiment. (**c**) Amount of 130 kDa NPAS3 detected in only those samples that showed more than mean levels of 130 kDa NPAS3. NPAS3 signal in each category is shown relative to the average NPAS3 signal over all samples. (**d**) Patient symptom severities, shown as total scores on the positive, negative and general psychopathology scales of PANSS [45], of schizophrenia patients with above background levels NPAS3 in blood serum (*n* = 7, “NPAS3 positive”) compared to other schizophrenia patients in the study (*n* = 41, “NPAS3 negative”). There are no significant differences between the two groups. (**e**) Average number of first- or second-degree relatives of these patients with known diagnosis of mental illness. * *p* < 0.05, ** *p* < 0.01, ns: not significant.

Specifically, signal detected by an anti-NPAS3 antibody was seen at low levels in many of the samples, while being significantly higher in a smaller proportion (Figure 4a, Supplementary Figure S7). A species of 130 kDa could be detected in many samples, but it was not clear in all instances, especially when the signal was extremely weak, if this represented a specific NPAS3 species, or was background noise (Figure 4b, no difference between diagnostic groups ANOVA, *p* = 0.56, F < F_crit_, df = 2, relative NPAS3 signal by diagnostic status: schizophrenia: 1.29 ± 0.51, depression: 0.97 ± 0.28, control: 0.73 ± 0.24). In order to focus only on signals that appeared specific, the data was re-examined, this time looking only at serum samples showing signal at 130 kDa that was above background levels (defined as being higher than the mean level of NPAS3 across all samples). This revealed a relationship between 130 kDa NPAS3 signal and diagnostic status (Figure 4c, ANOVA, *p* = 0.016, F > F_crit_, df = 2, relative NPAS3 signal by diagnostic status: schizophrenia: 9.24 ± 1.31, depression: 3.94 ± 0.49, control: 4.04 ± 0.55), with significantly higher NPAS3 in patients with schizophrenia than in control individuals or patients with depression (p_tukey_ = 0.003 in both cases). There was no difference between NPAS3 levels in depression patients and control individuals. It should be noted, however, that while the patients with schizophrenia were well matched in terms of sex and age to the control individuals, the population of patients with major depressive disorder were on average older, and with a higher proportion of females (see Supplementary Table S2 or details). It therefore appears that a subset of patients with schizophrenia express unusually high levels of NPAS3 in their blood serum, however, at this time, there is no evidence to suggest whether this directly ties to aggregation in the brain or elsewhere.

Of the seven schizophrenia patients with above background levels of NPAS3 in their blood serum (Figure 4c), all had a diagnosis of paranoid schizophrenia according to ICD-10 (code F20.0) [25]. Their symptom severity was not significantly higher than for 41 other patients with schizophrenia included in the study on the Positive and Negative Syndrome Scale (PANSS [45], multivariate ANOVA, *p* = 0.56, F < F_crit_, df = 3, Figure 4d, data was unavailable for the other two patients). Curiously, these patients on average had more first- or second-degree relatives with a dignosis of mental illness (1.00 ± 0.44) than the other 43 patients with schizophrenia included in the study (0.35 ± 0.10, one-way ANOVA, *p* = 0.022, df = 1, Figure 4e). Specifically, among the families of these seven patients, there were three cases of schizophrenia, one of major depressive disorder and three of alcohol dependence.

In addition to NPAS3, four other proteins have so far been implicated as aggregating in major mental illness: Collapsin Response Mediator Protein 1 (CRMP1) [18], Disrupted in Schizophrenia 1 (DISC1) [6], dysbindin-1 [17] and Trio and F-actin Binding Protein (TRIOBP) [19], all of which have previously detected as insoluble in the brains of schizophrenia patients. While NPAS3 was not seen in the insoluble fractions of the blood sera detected here, it was still possible that one or more of the other proteins might. We therefore probed our whole serum and insoluble serum fractions with antibodies against each of these four proteins. No clear immunoreactivity was seen to DISC1 or CRMP1, in either the whole serum or insoluble fraction. TRIOBP species, however, corresponding to both the TRIOBP-1 and TRIOBP-5/6 isoforms, were detected in several individuals (Supplementary Figure S8 and Table S5), but with no discernible relationship to diagnostic status, symptom severity or other epidemiological factors. Staining with an antibody against dysbindin-1 revealed two schizophrenia patients who appeared to have insoluble dysbindin-1 in their serum (Supplementary Figure S8 and Table S5).

## 4. Discussion

Proteinopathy is a well-established mechanism in neurodegenerative disease but has only recently been investigated in major mental illness [5]. While most of the proteins so far implicated as aggregating in chronic mental illnesses (schizophrenia in all cases plus, in some instances, also bipolar disorder and/or major depressive disorder) have been identified directly from patient brain samples [6,17,18,19], NPAS3 was implicated indirectly. Specifically, an ultrarare missense mutation, discovered in a family with schizophrenia and related disorders, was suggested to induce or enhance the aggregation propensity of NPAS3 [14,15].

In this first study to look for NPAS3 aggregation in brain tissue, although not in this case specifically in schizophrenia patients, we examined insular cortex samples from 40 individuals. The insular cortex has previously been implicated in various mental illnesses, including schizophrenia, and lies at the intersection of stimuli processing, decision making and emotional responses [46]. These samples were analyzed using the same insoluble protein purification technique that was previously employed to detect other aggregating proteins in major mental illness, based on the principle that aggregating proteins would normally be amongst the most insoluble proteins present in any tissue. While it was difficult to detect NPAS3 in the original homogenate samples (as might be expected for a principally neurodevelopmental protein) it was easily detected in the purified insoluble fractions, which were enriched for aggregating proteins. There was no clear relationship between total levels of insoluble NPAS3 in these samples and any specific diagnosis, although there was a trend towards higher levels in Alzheimer’s disease that warrants further study in a larger sample. It was striking, however, that insoluble NPAS3 was present in a very large proportion of these samples, far more than could be attributed to the presence of the previously reported V304I missense mutation [15]. This suggests that NPAS3 aggregation is a phenomenon beyond this individual family. It should be noted, however, that we did not look at brain samples from any patients with schizophrenia, the condition implicated in aggregation in the originally reported family [15], and so we cannot rule out the possibility that this insolubility/aggregation could be stronger in this condition than in the samples analyzed here.

Our studies in cultured human neuroblastoma cells appear to support this theory. While NPAS3 was generally seen in the nucleus, as expected, surprisingly some cells instead showed it to be also present in the cytoplasm (Figure 2, Supplementary Figures S2–S4). This was seen with both wild type and V304I mutant NPAS3 and corresponds to results obtained by isolating the insoluble fractions of cells expressing these proteins. For other nuclear proteins implicated as aggregating in diseases of the brain, such as TDP-43 and FUS, such failure to import protein into the nucleus is indicative of early stages of aggregation, in which misfolded and misassembled protein finds itself unable to pass through the nuclear pore [41,47]. The fact that, during insolubility experiments, a small proportion of over-expressed NPAS3 could be seen in the insoluble protein pellet, supports the idea that NPAS3 may behave in a similar manner to these proteins. Notably, application of oxidative stress to the neuroblastoma cells caused a significant reduction in cells that only displayed NPAS3 in the nucleus. This environmentally induced localization dwarfed any potential effect of the V304I mutation in this experimental setup. Therefore, while V304I may be a factor in NPAS3 aggregation, it is likely to be only one possible mechanism out of many. Indeed, by analogy with proteins that form aggregates in neurodegenerative conditions such as amyotrophic lateral sclerosis, it seems likely that a variety of mutations, spontaneous or familial, could impact NPAS3 aggregation, of which V304I is simply one. Environmental stresses, as simulated here, would also play a role. Sequencing of the *NPAS3* genes of these patients, and in particular those with NPAS3 in their serum and a family history of mental illness, would be an exciting next step in assessing the clinical relevance of these findings, although regrettably, this is not currently possible for us due to the ongoing COVID-19 pandemic.

In our mammalian cell experiments, we found that the critical domain for mislocalization was the PAS1 domain (aa 157-208). The presence of this was sufficient to prevent entry into the nucleus, and it led to the formation of visible aggregate-like structures in some cells. In contrast, previous studies showed NPAS3 protein aggregation to be dependent instead on the unstructured linker region between the two PAS domains (aa 211-331 [15]). This was based on an assay in which it was investigated whether or not recombinant NPAS3 proteins, treated with SDS, could enter an SDS-PAGE gel. While we did not clearly see this region to have an additive effect on whether these potential aggregates formed, we cannot discount the possibility that it might lead to aggregates forming in a more compacted manner, which could therefore show greater insolubility to SDS. There may also be differences because of the differing experimental systems used. Notably, our experiments were performed in a mammalian cell environment, although with the caveat that immortalized cells were used, and the NPAS3 fragments were still being expressed at non-physiological levels. The fact that the two constructs lacking the PAS1 domain showed similar results to each other, as did the two containing this domain, argues that the effects seen here are unlikely to be due to artifacts of the truncation points selected.

Interestingly, a frameshift mutation in NPAS3 has recently been reported at amino acid 214 in an individual with intellectual disability [48]. This mutation impairs the activity of NPAS3, and the mutant form is expressed at a lower level than the full-length protein (likely as a result of reduced stability) in human cultured cells [48]. The frameshift protein would be very close in length to the mislocalizing and aggregation-prone aa 1-208 fragment expressed in this study, indicating new mechanisms through which this mutation could cause disruption.

The presence of these N-terminal NPAS3 fragments in the nucleus was somewhat unexpected, as the canonical nuclear localization sequence (NLS) for NPAS3 is located in the C-terminal half of the protein [9], while the bHLH domain is predicted to have a nuclear export signal (NES) [28]. (It should be noted that this NES is present in all of the N-terminal fragments examined, and so should have an equal effect on each). Analysis using NLStradamus [49] shows a potential second NLS in NPAS3, within the bHLH at amino acids 53–65. While the prediction for this is much weaker than for the canonical NLS, it nevertheless would explain why these proteins are present in the nucleus, either exclusively or also in the cytoplasm. Conversely, the fact that a major NLS is missing would potentially explain the greater average number of cells displaying NPAS3 in the cytoplasm, compared to experiments using the full-length protein (Figure 3). The addition of the PAS1 domain appears to block this nuclear localization, presumably because aggregated or proto-aggregating NPAS3 cannot be transported into the nucleus, as is the case for other disease-related aggregating proteins, such as TDP-43 [41]. We also looked at constructs that were truncated from the N-terminus: encoding the C-terminal activation domain (TAD), or the TAD plus the two PAS regions (Supplementary Figure S9). The protein fragments encoded were consistently found in the nucleus, in agreement with previous data [28]. Potentially this could be because the more potent NLS in the TAD, combined with the lack of an NES, masks the effect of the NLS near to the N-terminus. Alternative explanations include a stabilizing effect of PAS1 or the requirement for additional features N-terminal of PAS1 for blocking of its uptake into the nucleus. We also attempted to rescue mislocalization of N-terminal NPAS3 fragments by co-expressing them with full-length NPAS3. Despite the fact that these fragments should include dimerization domains [50] there was no indication of rescue (Supplementary Figure S10), indicating that either these fragments cannot form homodimers, or that these fragments are unable to enter the nucleus, even when bound to NPAS3 species with the more potent NLS.

We also detected NPAS3 in the blood serum of individuals, being more prominent in a subset of individuals with schizophrenia. NPAS3 is not reported to be an extracellular or secreted protein and is canonically found only in the cell nucleus [7,28]. It is, therefore, more likely that this NPAS3 originates from the apoptosis or autophagy of other cells. The source of cells that produced this serum NPAS3 is currently unknown. Canonically, NPAS3 is considered a protein of the brain, which is where NPAS3 transcripts are principally seen [7,44], however, NPAS3 protein is present in many tissues (Human Protein Atlas, https://www.proteinatlas.org/ENSG00000151322-NPAS3/tissue (accessed on 1 October 2021), [35]). It is unlikely that the brain represents the source of this protein, due to the blood-brain barrier. The next most prominent expression site of *NPAS3* expression, based on data in the Human Protein Atlas, are tissues of the reproductive systems, however, this also appears unlikely as NPAS3 was seen in the serum of both males and females. Other potential sources of NPAS3 based on this data include the colon, thyroid or pituitary gland.

It is not currently clear whether the presence of NPAS3 in the blood of certain patients is related in any way to aggregation, although it is plausible that disrupted NPAS3 homeostasis in the brain could correlate with similar disruptions elsewhere, particularly if they arise from genetic variation. It is curious that the presence of this NPAS3 in the blood correlated only with schizophrenia, for which NPAS3 aggregation was implicated, but not with major depressive disorder [15]. This may correlate with the lack of specific NPAS3 aggregation in insular cortex samples patients with major depressive disorder and victims of suicide, seen here. It also leaves open the possibility that aggregation may still be more prominent in the brains of at least some patients with schizophrenia. The control population in the blood study, however, was more closely matched to the patients with schizophrenia than those with major depressive disorder, the latter having more females and a higher average age. We therefore cannot rule out the possibility that a more closely matched set of controls may have revealed a significant effect on serum NPAS3 in major depressive disorder. The low numbers of patient blood samples that clearly showed the presence of NPAS3, while inconvenient diagnostically, is nevertheless not a total surprise, as genetic studies have indicated schizophrenia to be biologically heterogenous, with only variants of very low effect being found consistently across wide patient populations. It would be extremely interesting to know to investigate in a larger sample, whether high levels of serum NPAS3 correlate either with specific subtypes of schizophrenia, or with the severity of specific symptoms.

## 5. Conclusions

Aggregation of NPAS3, as assayed by looking for it a purified insoluble fraction of insular cortex homogenates, appears to be a far more widespread phenomenon than the previously reported V304I familial mutation in NPAS3. Assays in cell culture indicate that it can also be induced by more general oxidative stress, and it seems plausible that, as in the case with proteinopathy in neurodegenerative disorders, a combination of genetic and environmental factors contribute to it. While aggregation of NPAS3 occurs in many individuals, study is now needed to determine if it is more generally enhanced in schizophrenia patients, as well as if this correlates with altered levels of NPAS3 detected in the blood.

## Supplementary Materials

The following are available online at https://www.mdpi.com/article/10.3390/jpm11111070/s1, Table S1: Demographic information on the participants of the brain study, Table S2: Demographic information on the participants of the blood serum study, Table S3: Sources and generation of plasmids used in this study, Table S4: Primers used for cloning in this study, Table S5: TRIOBP-1 and dysbindin in blood serum, Figure S1: Confirmation of antibodies, used to test clinical samples, by Western blotting, Figure S2: Additional analysis of full length NPAS3 localization, Figure S3: Additional images of full length NPAS3 in cell culture over 24–72 h, Figure S4: Expression of wild type NPAS3 from alternative vectors, Figure S5: A small portion of NPAS3 is insoluble when expressed in cell culture, Figure S6: Additional analysis of N-terminal NPAS3 fragment localizations, Figure S7: NPAS3 in additional patient serum samples, Figure S8: TRIOBP-1 and dysbindin in blood serum, Figure S9: Expression of N-terminally truncated NPAS3 fragments, Figure S10: Co-transfection of N-terminal fragments of NPAS3 with full length NPAS3.
