# Supplementary material for: Protein Aggregation of NPAS3, Implicated in Mental Illness, Is Not Limited to the V304I Mutation
Source: J Pers Med. 2021 Oct 23;11(11):1070. doi: 10.3390/jpm11111070 (PMC8623263; doi:10.3390/jpm11111070)
Supplement: Supplementary file 1 [file jpm-11-01070-s001.zip › jpm-1390712-supplementary.pdf]

Protein aggregation of NPAS3, implicated in mental illness, is not limited to the V304I mutation

Bobana Samardžija*, Aristea Pavešić Radonja*, Beti Zaharija, Mihaela Bergman, Éva Renner, Miklós Palkovits, Gordana Rubeša and Nicholas J. Bradshaw

Supplementary information

Supplementary Table S1: Demographic information on the participants of the brain study

Supplementary Table S2: Demographic information on the participants of the blood serum study

Supplementary Table S3: Sources and generation of plasmids used in this study

Supplementary Table S4: Primers used for cloning in this study

Supplementary Table S5: TRIOBP-1 and dysbindin in blood serum

Supplementary Figure S1: Confirmation of antibodies, used to clinical samples, by Western blotting

Supplementary Figure S2: Additional analysis of full length NPAS3 localization

Supplementary Figure S3: Additional images of full length NPAS3 in cell culture over 24-72 hours

Supplementary Figure S4: Expression of wild type NPAS3 from alternative vectors

Supplementary Figure S5: A small portion of NPAS3 is insoluble when expressed in cell culture

Supplementary Figure S6: Additional analysis of N-terminal NPAS3 fragment localizations

Supplementary Figure S7: NPAS3 in additional patient serum samples

Supplementary Figure S8: TRIOBP-1 and dysbindin in blood serum

Supplementary Figure S9: Expression of N-terminally truncated NPAS3 fragments

Supplementary Figure S10:Co-transfection of N-terminal fragments of NPAS3 with full length NPAS3

*Equal first author contribution

Group	Sex (number, percent)		Age (years)			Post mortem interval (hours)	
	Male	Female	Mean	SD	p_{tukey} vs. control	Mean	SD
Suicide victim (n = 16)	10 (71%)	4 (29%)	46.77	14.69	0.012	4.40	1.51
Control (n = 18)	9 (64%)	5 (36%)	61.89	11.25	N/A	4.11	1.94
Major depression disorder (n = 6)	2 (33%)	4 (67%)	76.17	19.4	0.144	7.17	1.73
Alzheimer's (n = 6)	3 (50%)	3 (50%)	77.00	12.54	0.111	4.50	1.05

Supplementary Table S1: Demographic information on the individuals included in the brain study. SD: standard deviation. There was no overall difference in sex between the samples ($\chi^2 = 2.897$, $p = 0.408$, df = 3). Differences between diagnostic categories were analyzed using one-way ANOVA, showing a significant effect of age ($p < 0.001$, F = 10.999, df = 3), but not of post mortem interval ($p = 0.640$, F = 0.567, df = 3). Tukey's post hoc test was therefore used to investigate age differences between control individuals and each of the patient categories (values shown in the table).

Group	Sex (number, percent)			Age (years)		
	Male	Female	χ^2 p value vs control	Mean	SD	P_{tukey} vs control
Schizophrenia (n = 50)	34 (68%)	16 (32%)	0.404	47.20	11.03	0.748
Major depressive disorder (n = 50)	15 (30%)	35 (70%)	0.003	58.36	11.18	< 0.001
Control (n = 50)	30 (60%)	20 (40%)	N/A	48.74	9.94	N/A

Supplementary Table S2: Demographic information on the individuals included in the blood serum study. SD: standard deviation. There is a significant difference in the proportion of each sex in the samples ($\chi^2 = 2.897$, $p = 0.408$, df = 3). Pairwise comparisons were therefore made versus control individuals (df = 1) and are shown in the table. Differences between diagnostic categories were analyzed using one-way ANOVA, showing a significant effect ($p < 0.001$, F = 8.498, df = 2). Tukey's post hoc test was therefore used to investigate age differences between control individuals and each of the patient categories (values shown in the table).

Plasmid	Vector backbone	Gene insert	Origin
1	pENTR1A no ccDB	(None)	Addgene, clone 17398 - Campeau et al (2009) PLOS One 4:e6529
2	pdcDNA-FlagMyc	(Gateway cassette)	BCCM/LMBP Plasmid Collection, clone LMBP 4705
3	pDEST-CMV-N-mCherry	(Gateway cassette)	Addgene, clone 123215 – Agrotis et al (2019) Autophagy 15:976-997
4	pETG10A	(Gateway cassette)	Gift from EMBL, Protein Expression & Purification Core Facility
5	pCI-HA	NPAS3 WT	Gift from Fred Berry - Luoma & Berry (2018) BMC Mol. Biol. 19:14
6	pCI-HA	NPAS3 V304I	Gift from Fred Berry - Luoma & Berry (2018) BMC Mol. Biol. 19:14
7	pCI-HA	NPAS3 (116-933)	Gift from Fred Berry - Luoma & Berry (2018) BMC Mol. Biol. 19:14
8	pCI-HA	NPAS3 (451-933)	Gift from Fred Berry - Luoma & Berry (2018) BMC Mol. Biol. 19:14
9	pENTR223	Dysbindin 1A	DNASU Plasmid Repository, clone HsCD00510719
10	pENTR223.1	NPAS3	DNASU Plasmid Repository, clone HsCD00080332
11	pENTR1A	NPAS3 (1-111)	Subcloned from plasmid 4 with primers A & B, then F & G. Ligated into the *KpnI* and *EcoRI* sites of plasmid 1
12	pENTR1A	NPAS3 (1-156)	Subcloned from plasmid 4 with primers A & C, then F & G. Ligated into the *KpnI* and *EcoRI* sites of plasmid 1
13	pENTR1A	NPAS3 (1-208)	Subcloned from plasmid 4 with primers A & D, then F & G. Ligated into the *KpnI* and *EcoRI* sites of plasmid 1
14	pENTR1A	NPAS3 (1-354)	Subcloned from plasmid 4 with primers A & E, then F & G. Ligated into the *KpnI* and *EcoRI* sites of plasmid 1
15	pdcDNA-FlagMyc	NPAS3	LR clonase recombination of plasmids 2 and 10
16	pdcDNA-Flag	NPAS3 (1-111)	LR clonase recombination of plasmids 2 and 11
17	pdcDNA-Flag	NPAS3 (1-156)	LR clonase recombination of plasmids 2 and 12
18	pdcDNA-Flag	NPAS3 (1-208)	LR clonase recombination of plasmids 2 and 13
19	pdcDNA-Flag	NPAS3 (1-354)	LR clonase recombination of plasmids 2 and 14
20	pDEST-CMV-N-mCherry	(None - control)	LR clonase recombination of plasmids 1 and 3
21	pDEST-CMV-N-mCherry	NPAS3	LR clonase recombination of plasmids 3 and 10
22	pETG10A	Dysbindin 1A	LR clonase recombination of plasmids 4 and 9
23	pETG10A	NPAS3 (1-208)	LR clonase recombination of plasmids 4 and 13
24	pETG10A	TRIOBP-1 (281-466)	Gift from Carsten Korth - Bradshaw et al (2017) J. Biol. Chem. 292:9583-9598

Supplementary Table S3: Plasmids used in this study, and their source or generation. For details of the primers referred to, see supplementary table S3.

Primer	Name	Sequence
A	NPAS3 1 F	GAAGTCGACATGGCGCCCAC
B	NPAS3 111 R	GAAGAATTCTCATCAGTCCCCCTGG
C	NPAS3 156 R	GGCGAATTCTCATCACTGCAAAATGTGG
D	NPAS3 208 R	GGCGAATTCTCATCACTCAGCCATC
E	NPAS3 354 R	GGGGCGAATTCTCATCACATATAATCACTAATC
F	Extension F	GCTATAAGGATCCGGTACCTAGTCGACATG
G	Extension R	GGCACCAGCTCGAGTCTAGAATTCTCATC

Supplementary Table S4: Primers used for cloning in this study.

Protein species	Schizophrenia	Depression	Control
TRIOBP-5/6 (approx. 250 kDa)	3/50 (6%)	2/50 (4%)	2/50 (4%)
TRIOBP-1 (approx. 70 kDa)	2/50 (4%)	1/50 (2%)	1/50 (2%)
Dysbindin-1A (approx. 40 kDa)	2/50 (4%)	0/50 (0%)	0/50 (0%)

Supplementary Table S5: Number of individual insoluble protein fractions from blood serum showing immunoreactivity for TRIOBP and dysbindin samples, by diagnostic status

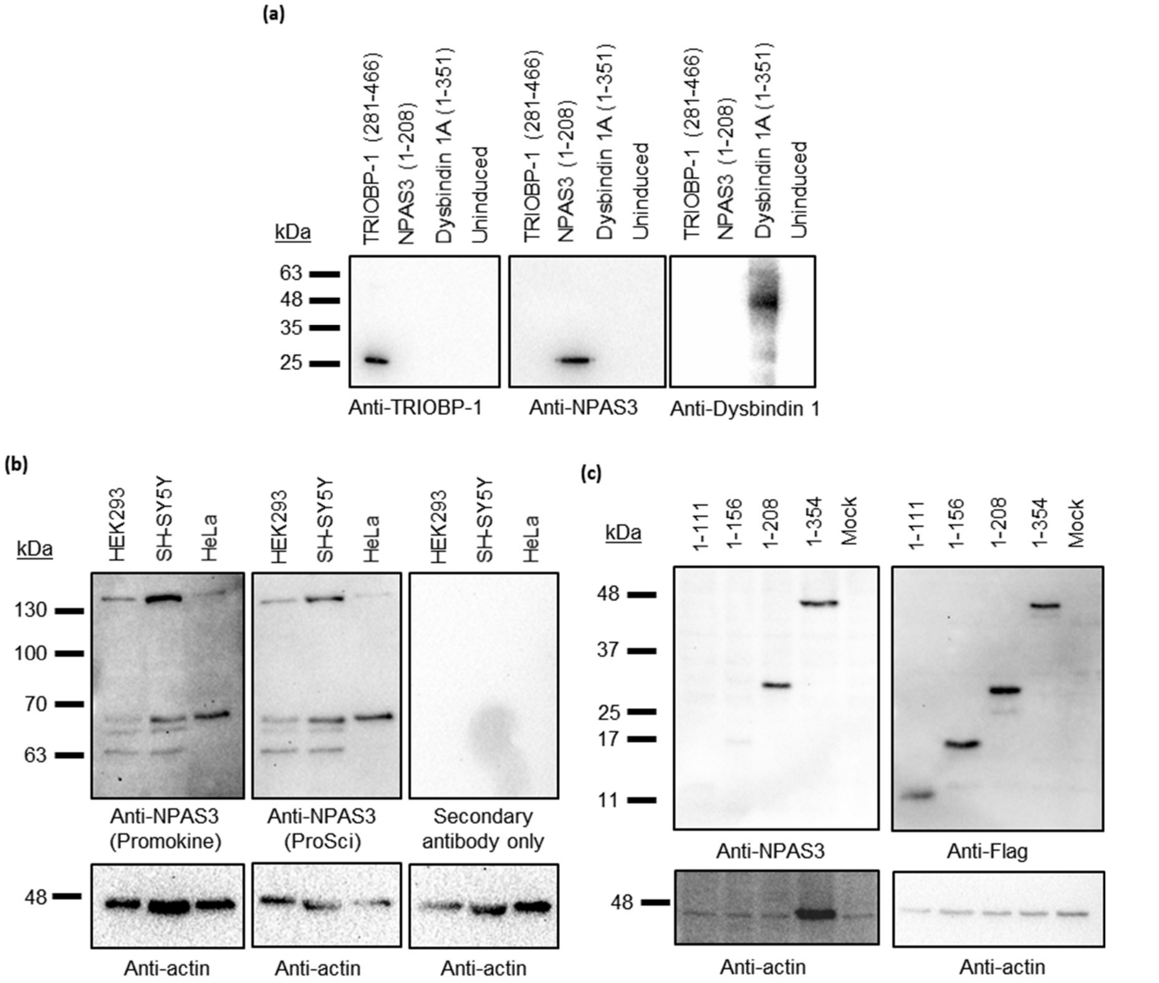

Supplementary Figure S1. Confirmation of antibodies, used to test clinical samples, by Western blotting. In all images, blots arranged vertically indicate re-staining of the same membrane, while blots arranged horizontally represent identical samples, run, blotted and stained in parallel. (**a**) Lysates from bacteria expressing recombinant proteins: amino acids 281-466 of human TRIOBP-1 (its central coiled coil domain), amino acids 1-208 of human NPAS3 (its basic helix-loop-helix and PAS1 domains) or the entirety of human dysbindin-1A. Uninduced bacteria are shown as a negative control. Blots were probed with the three antibodies used to detect TRIOBP, NPAS3 and dysbindin-1 in serum samples. Each antibody only recognizes the recombinant protein that it is expected to. (**b**) Endogenous NPAS3 in cell line lysates. The Promokine antibody is the anti-NPAS3 antibody used in all other experiments of this manuscript, and detects both full length (130+ kDa) NPAS3 plus a variety of processed species (63-70 kDa) in each lysate, with slight differences in each cell line. Another commercial antibody against NPAS3 (from ProSci) recognizes exactly the same banding pattern, implying both antibodies to be detecting the same protein. No bands were seen using secondary antibody only. (**c**) HEK293 lysates, expressing Flag-tagged N-terminal fragments of NPAS3 (amino acid numbers shown). While an anti-Flag antibody can detect all four fragments, the anti-NPAS3 antibody used for experiments in this paper shows significant enrichment for the larger two fragments, implying its major epitope(s) to be within amino acids 157-208. Note that actin stating was done after NPAS3/Flag staining, and there is anti-NPAS3 bleed through in the fourth lane of the bottom left panel.

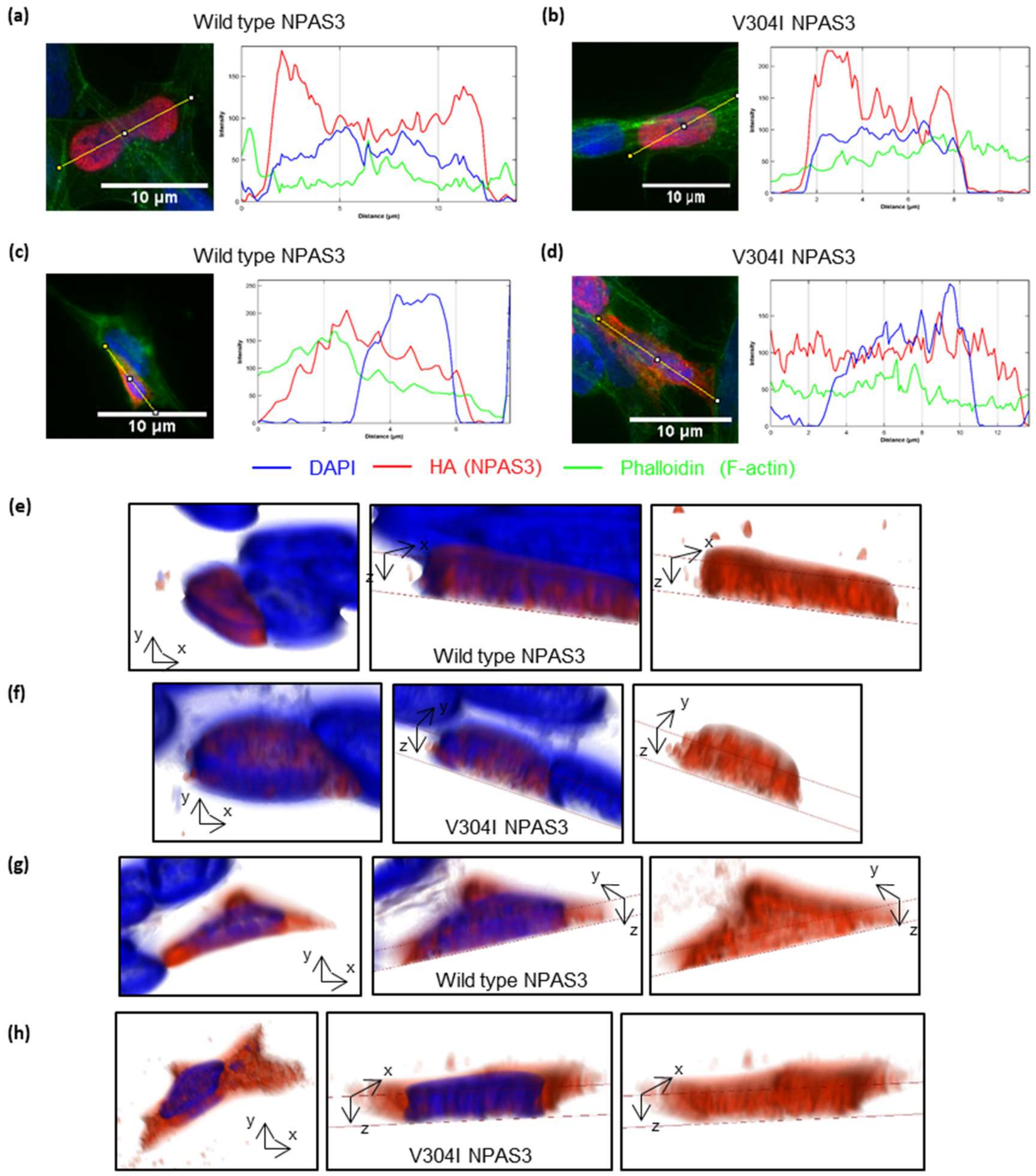

Supplementary Figure S2: Additional analysis of full length NPAS3 localization. **(a-d)** Quantification of DAPI (nuclear), HA (NPAS3) and phalloidin (actin) signal along a one-dimensional line, taken from a single focal plane, for images used in figure 2c-f of the main text. For both WT and V304I NPAS3, there are cells in which the HA signal clearly corresponds to the location of the DAPI signal (a,b), demonstrating NPAS3 to be in the nucleus. For a minority of cells examined, level of HA is comparable in locations with and without DAPI (c,d), indicating that NPAS3 is found in the cytoplasm, in addition to the nuclear. **(e-h)** 3-D representations of cells, compiled from images in multiple focal planes using CellSens software (Olympus). For each cell, the leftmost image shows the x-y plane, while the remaining two show a cut through the z-axis. SH-SY5Y cells are shown expressing WT and V304I NPAS3 (in red, anti-HA antibody), along with DAPI (blue). The central and righthand images for each cell are identical apart from the inclusion or exclusion of DAPI signal. For both constructs, the majority of cells show NPAS3 to be predominantly or exclusively in the nucleus (e,f), while a minority of cells show it to in the cells body as well.

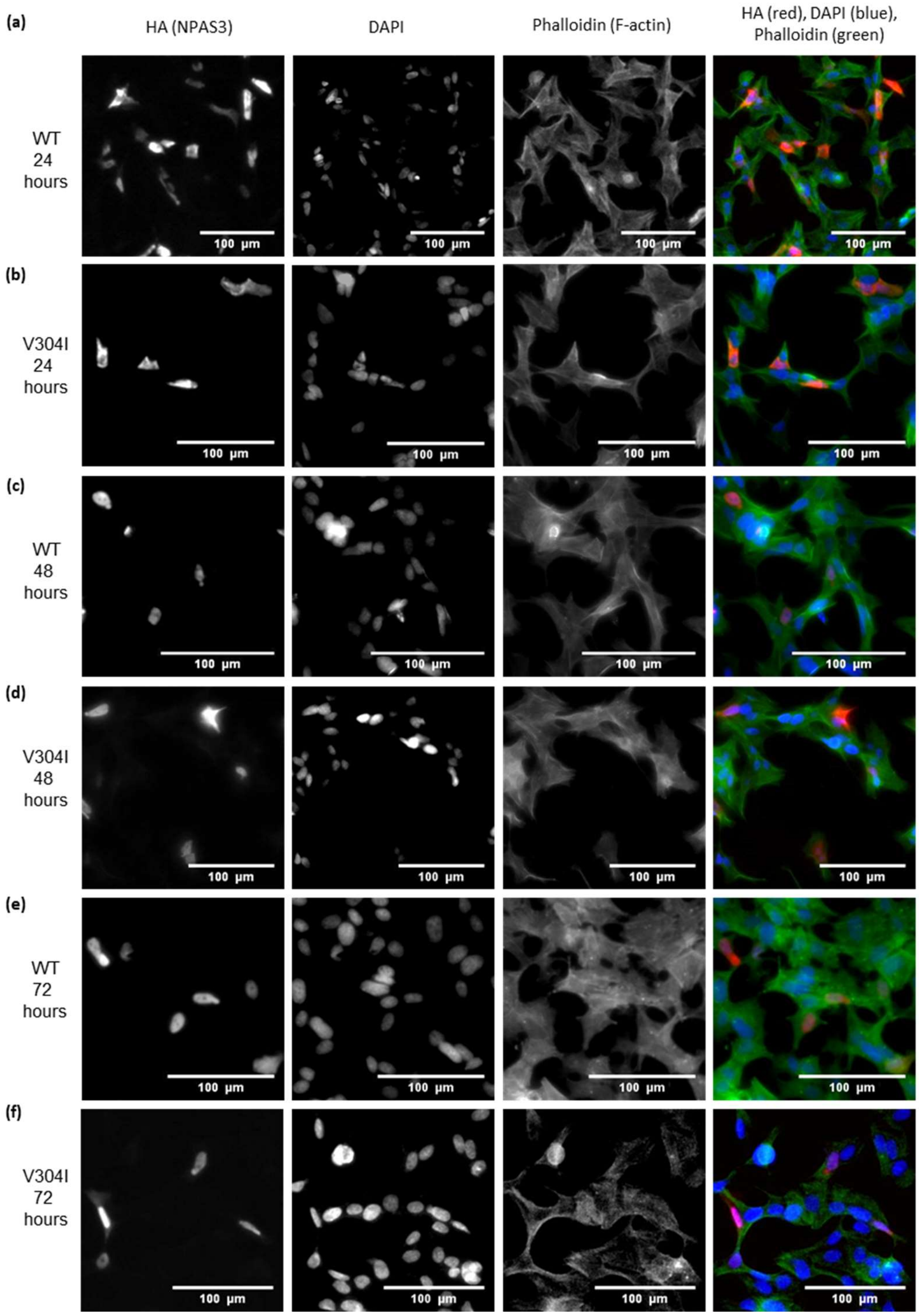

Supplementary Figure S3: Additional images of full length NPAS3 in cell culture over 24-72 hours**.** SH-SY5Y are shown, transfected with HA-tagged full length NPAS3 that is either wild type (WT) or has the V304I mutation, shown at a wider magnification to allow viewing of multiple cells. No consistent differences in expression *(Supplementary figure S2, continued)* pattern are seen between the two constructs, when fixed and viewed 24, 48 or 72 hours after transection. (**a**) Wild type NPAS3 after 24 hours. (**b**) V304I mutant NPAS3 after 24 hours. (**c**) Wild type NPAS3 after 48 hours. (**d**) V304I mutant NPAS3 after 48 hours. (**e**) Wild type NPAS3 after 72 hours. (**f**) V304I mutant NPAS3 after 72 hours.

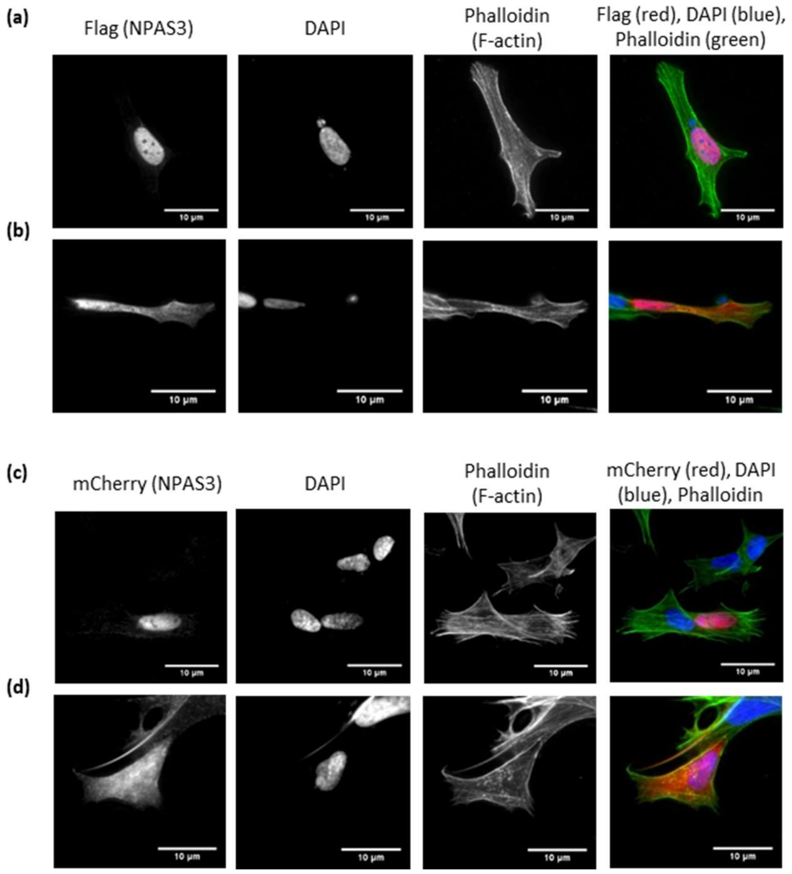

Supplementary Figure S4: Expression patterns of full length, human, wild type NPAS3 in SH-SY5Y cells, when expressed from two alternative plasmid vectors, which add either a Flag tag (**a**,**b**) or an mCherry fusion protein (**c**,**d**). In both instances, the expressed NPAS3 is seen predominantly in the nucleus (**a**,**c**), while in approximately 10% of cells it is instead seen both in the nucleus and the cytoplasm (**b**,**d**).

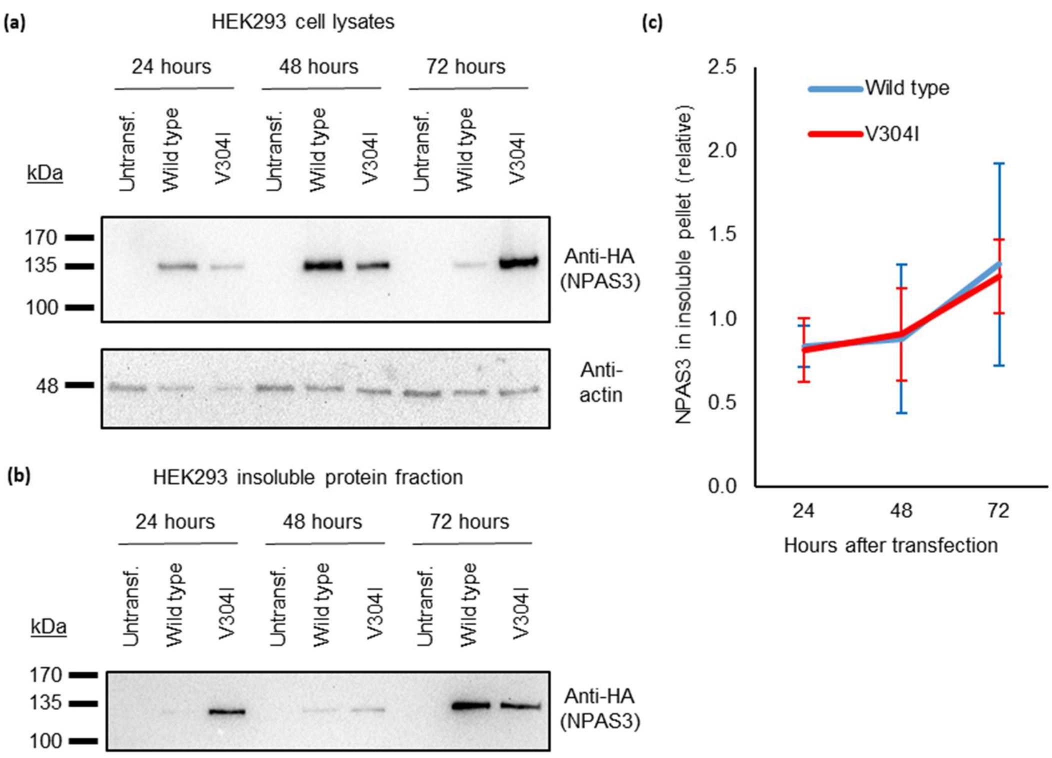

Supplementary Figure S5: A small portion of NPAS3 is insoluble when expressed in cell culture. HEK293 cells were transfected with HA-tagged wild type of V304I mutant NPAS3, or left transfected as a control. Cells were lysed 24, 48 or 72 hours after transfection (**a**) Expression of NPAS3 in the whole cell lysates. (**b**) Expression of NPAS3 in the purified insoluble protein pellet of these cells (enriched 10-fold), shows that a proportion of each protein is consistently found to be insoluble, implying aggregation. (**c**) Quantification of NPAS3 (HA) in the insoluble pellet shows no constant differences between the wild type and V304I mutant NPAS3 over three independent experiments.

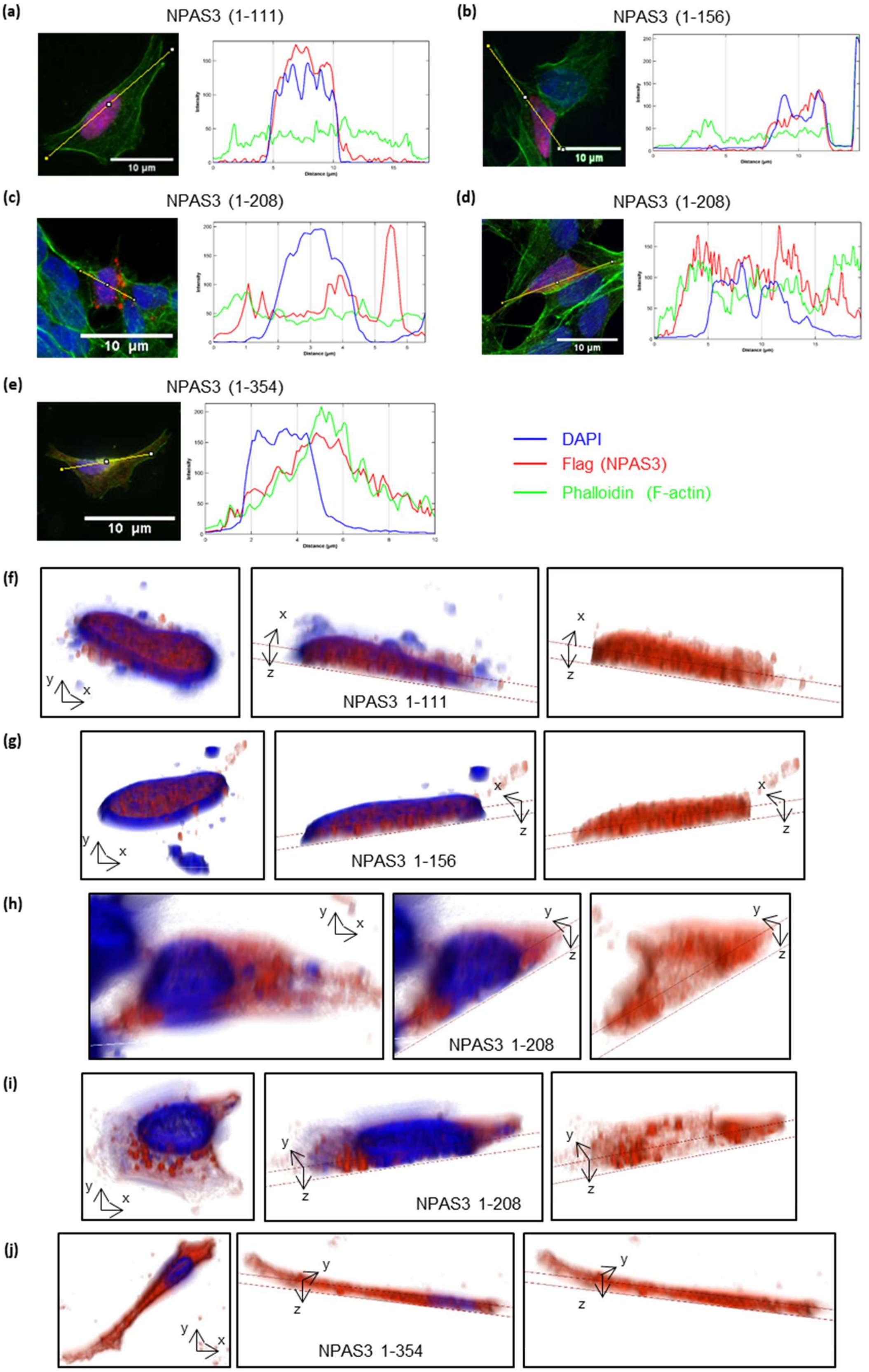

(a)
NPAS3 (1-111)
10 µm
(b)
NPAS3 (1-156)
10 µm
(c)
NPAS3 (1-208)
10 µm
(d)
NPAS3 (1-208)
10 µm
(e)
NPAS3 (1-354)
10 µm
DAPI
Flag (NPAS3)
Phalloidin (F-actin)
(f)
NPAS3 1-111
(g)
NPAS3 1-156
(h)
NPAS3 1-208
(i)
NPAS3 1-208
(j)
NPAS3 1-354

Supplementary Figure S6 (previous page)**:** Additional analysis of the localization of N-terminal fragments of NPAS3. **(a-d)** Quantification of DAPI (nuclear), Flag (NPAS3 fragments) and phalloidin (actin) signal along a one-dimensional line, taken from a single focal plane, for images used in figure 3c-g of the main text. For both the fragment containing amino acids 1-111 (a) and 1-156 (b) of NPAS3, the Flag signal clearly corresponds to the location of the DAPI signal (a,b), demonstrating the fragment to be in the nucleus. For the constructs containing amino acids 1-208 (c,d) and 1-354 (e) of NPAS3, the level of Flag is comparable or lower when DAPI is present, demonstrating these to be present in the cytoplasm, either in addition to or instead of the nucleus. **(e-i)** 3-D representations of cells, compiled from images in multiple focal planes using CellSens software (Olympus). For each cell, the leftmost image shows the x-y plane, while the remaining two show a cut through the z-axis. SH-SY5Y cells are shown expressing fragments of NPAS3 including amino acids 1-111 (e), 1-156 (f), 1-208 (g,h) and 1-354 (j) (all in red, anti-Flag antibody), along with DAPI (blue). The central and righthand images for each cell are identical apart from the inclusion or exclusion of DAPI signal. For fragments 1-111 and 1-156, the majority of cells show NPAS3 to be predominantly or exclusively in the nucleus, while 1-208 and 1-354 are prominently in the cytoplasm, with limited nuclear signal.

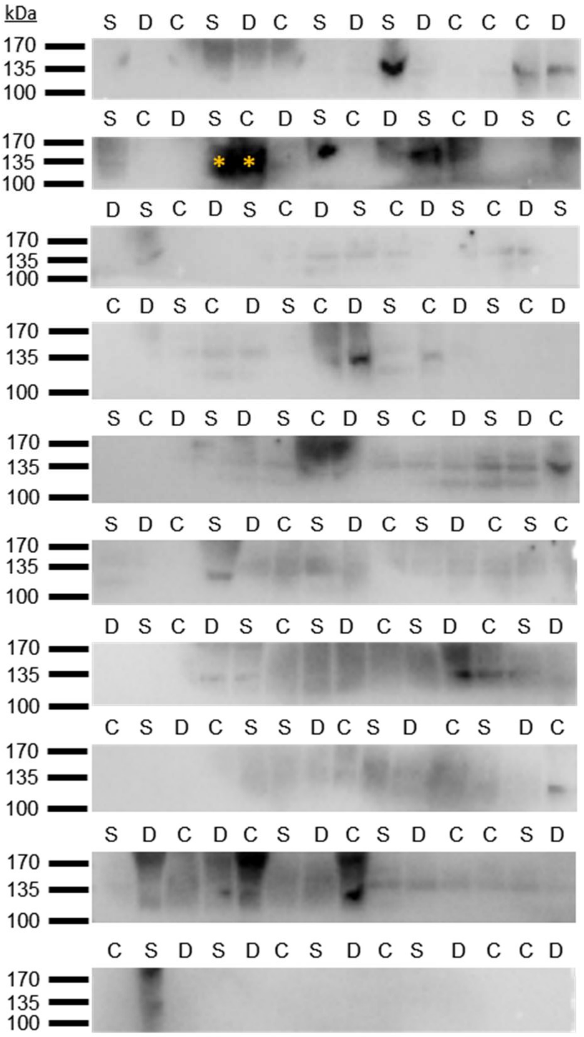

Supplementary Figure S7: NPAS3 in the serum of patients with schizophrenia (S) and major depressive disorder (D), as well as control individuals (C). Signal at around the 135 kDa mark was analyzed further (figures 1b and 1c), based on the known size of NPAS3 (for example in figure 3b and supplementary figure S5b). The two samples marker with orange asterisks were not included in the analysis in figures 1b&c as the amorphous nature of the signal did not appear to represent a protein species.

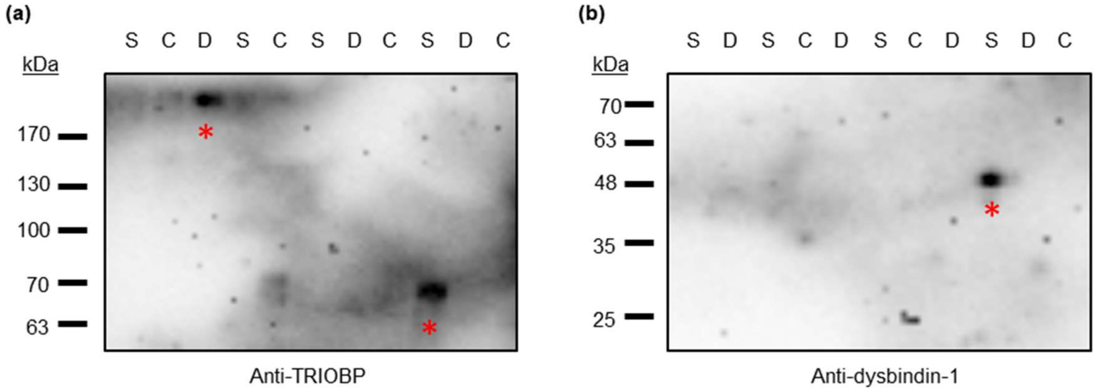

Supplementary Figure S8: Western blots showing examples of the insoluble (aggregate) fractions of blood serum from patients with schizophrenia (S) or major depressive disorder (D), as well as control individuals (C). (**a**) Staining with anti-TRIOBP, of expected molecular weights 68-74 kDa (TRIOBP-1 isoform, exact size depends on alternate start codon usage [15]), 243 kDa (TRIOBP-5) and 261 kDa (TRIOBP-6). (**b**) Staining with anti-Dysbindin-1, of expected molecular weights 40 kDa, 35 kDa and 30 kDa (Dysbindin-1A, B and C isoforms respectively). Bands considered positive are indicated with red asterisks. Each lane was loaded with 10 µl of insoluble serum fraction (purified from 167 µl of crude serum).

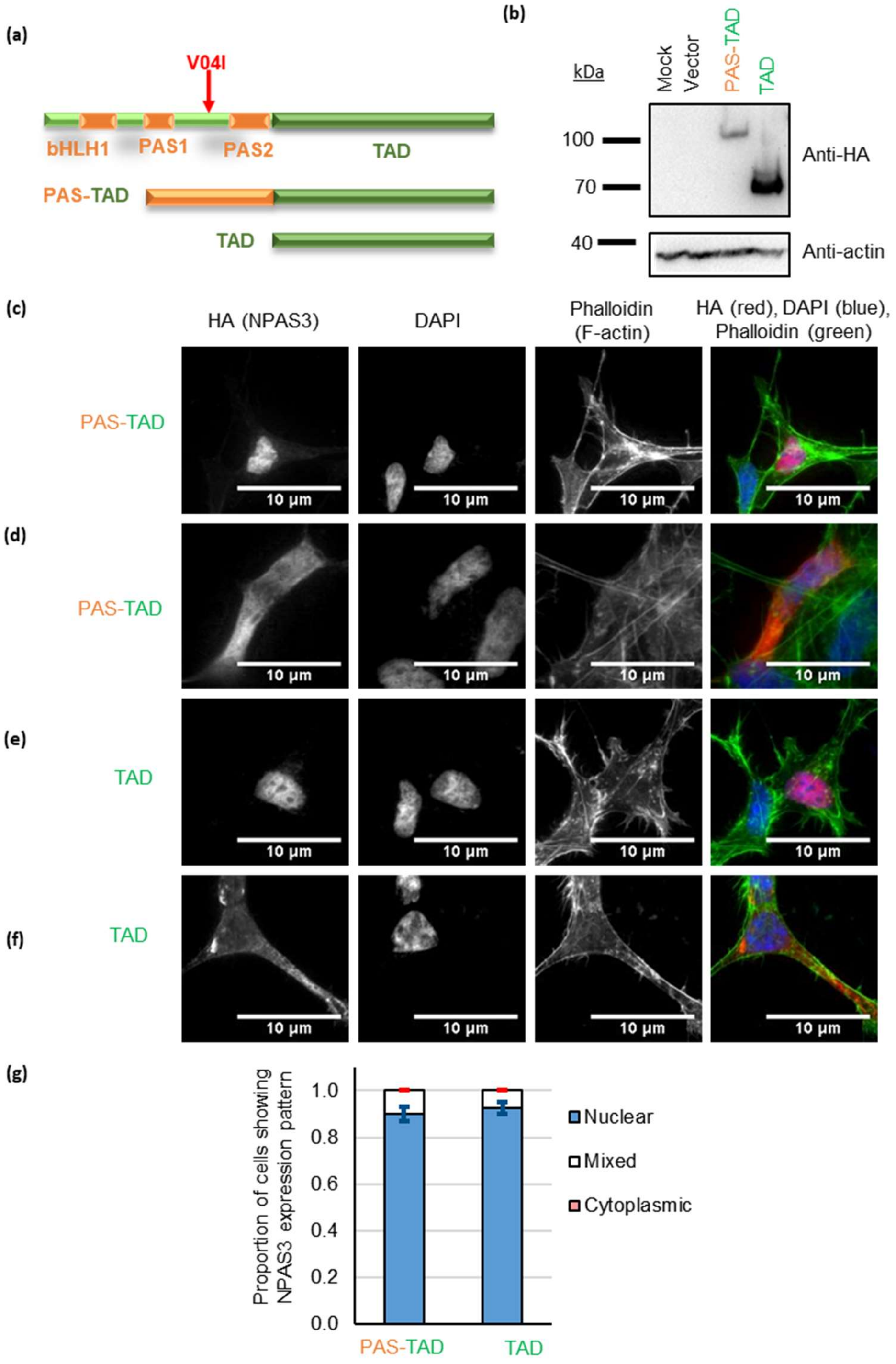

Supplementary Figure S9: Subcellular localizations of H-terminally truncated NPAS3 fragments with HA tags in SH-SY5Y cells. (**a**) Schematic of the fragments of NPAS3 expressed. (**b**) Expression of the HA-tagged NPAS3 fragments confirmed by Western blotting in HEK293 cells. A mock transfection with no plasmid and transfection with an empty vector are used as negative controls. (**c-g**) Typical localization patterns of these NPAS3 fragments when over-expressed in SH-SY5Y cells: fragments containing both the PAS and TAD domains (**c**,**d**) or the TAD domain alone. The nucleus (DAPI) and actin cytoskeleton (phalloidin) are shown for context. (**g**) Quantification of the localization patterns of these aggregates in a blinded assay (5-7 experimental replicates, with 9-10 cells being counted in each).

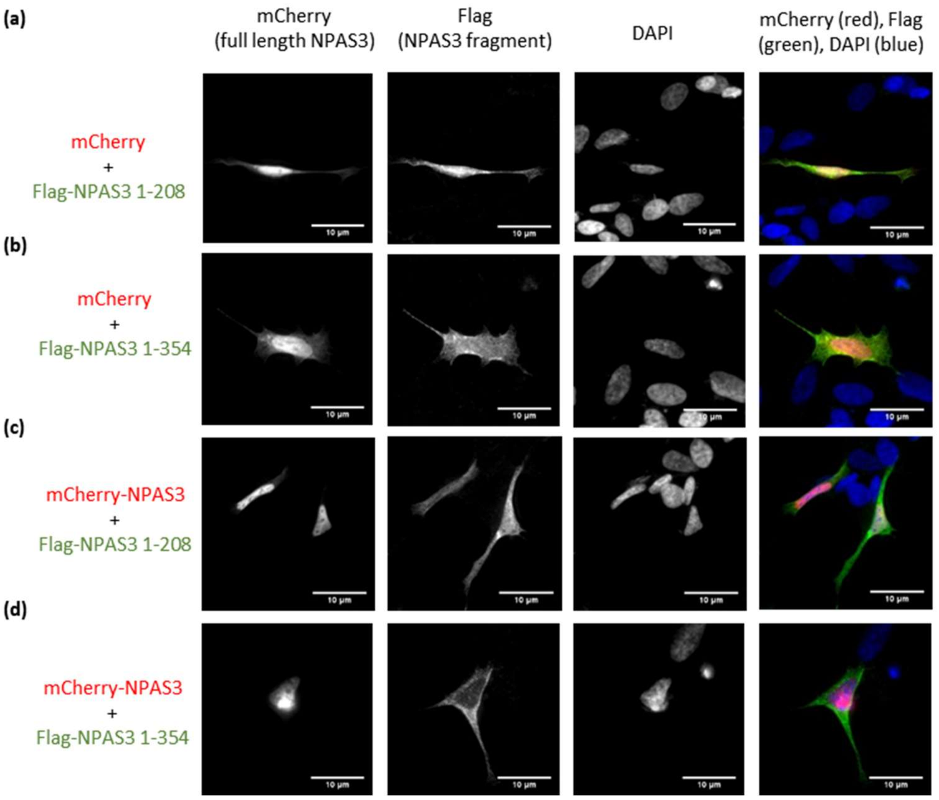

Supplementary Figure S10: SH-SY5Y cells co-expressing N-terminal fragments of NPAS3 (Flag tagged) with full length NPAS3 (fused to mCherry). Constructs expressing amino acids 1-208 (**a**,**c**) or 1-354 (**b**,**d**) of NPAS3 were co-transfected with plasmids including either mCherry alone (**a**,**b**), or mCherry fused to full length NPAS3 (**c**,**d**). In all instances, the fragment of NPAS3 remained prominently in the cytoplasm, with no obvious increase in nuclear localization when co-transfected with full length NPAS3. Similarly, full length NPAS3 was seen in the nucleus as expected.
